# Supplementary material for: Asynchronous magnetic resonance elastography: Shear wave speed reconstruction using noise correlation of incoherent waves
Source: Magn Reson Med. 2022 Oct 27;89(3):990–1001. doi: 10.1002/mrm.29502 (PMC9792433; doi:10.1002/mrm.29502)
Supplement: Supplementary file 1 — DATA S1 MATLAB code used to generate the numerical simulations of Figure 1 [file MRM-89-990-s002.zip › k-Wave/helpfiles/example_ivp_opposing_corners_sensor_mask.html]

Defining A Sensor Mask By Opposing Corners Example (k-Wave)


# Defining A Sensor Mask By Opposing Corners Example

This example demonstrates how to define a sensor mask using the grid coordinates of two opposing corners of a rectangle. It builds on the Homogeneous Propagation Medium  and Using A Binary Sensor Mask examples.

- Open the file in the MATLAB Editor
- Run the file in MATLAB

## Contents

- Defining a sensor mask by opposing corners
- Indexing the output data

## Defining a sensor mask by opposing corners

In addition to the Cartesian and binary sensor masks used in the previous two examples, it is also possible to define a sensor mask using the grid coordinates of two opposing corners of a line (1D), rectangle (2D), or cuboid (3D). The recorded data is identical to that recorded using a binary sensor mask covering the same region, however, the output is indexed differently as discussed below. In 2D, the rectangular regions are specified as column vectors in the form `[X1; Y1; X2; Y2]`, where `(X1, Y1)` and `(X2, Y2)` define two opposing corners of the desired rectangular sensor region in units of grid points, where `(1, 1)` defines the upper left corner of the grid. Multiple rectangles can be specified by adding additional column vectors to `sensor.mask` as shown below.

```
% define the first rectangular sensor region by specifying the location of
% opposing corners
rect1_x_start = 25;
rect1_y_start = 31;
rect1_x_end = 30;
rect1_y_end = 50;

% define the second rectangular sensor region by specifying the location of
% opposing corners
rect2_x_start = 71;
rect2_y_start = 81;
rect2_x_end = 80;
rect2_y_end = 90;

% assign the list of opposing corners to the sensor mask
sensor.mask = [rect1_x_start, rect1_y_start, rect1_x_end, rect1_y_end;...
               rect2_x_start, rect2_y_start, rect2_x_end, rect2_y_end].';
```

The computation is again started by calling `kspaceFirstOrder2D`. A snapshot of the simulation showing the two rectangular regions is given below.


## Indexing the output data

When the sensor mask is defined using opposing corners, the output data is always returned as a structure (in the same way as when `sensor.record` is defined as discussed in the Recording The Particle Velocity Example). In this case, the recorded time-varying acoustic pressure is accessed as `sensor_data(rect_num).p`, where `rect_num` is the number of the rectangular region within `sensor.mask`. In this example, two rectangular regions are assigned to `sensor.mask`, so `rect_num` would be set to `1` or `2`. As the shape of the sensor region is always regular, the output data is also indexed differently compared to using a Cartesian or binary sensor mask. In 2D, the sensor data is indexed `sensor_data(x_index, y_index, time_index)` (similarly in 1D and 3D). This makes it particularly useful for recording the results of a simulation in a plane or line without needing to reshape the output data. If the regions are overlapping, the overlapped data is returned as part of both sensor regions. An example of querying the size of the sensor data is shown below.

```
>> size(sensor_data(1).p)

ans =

     6    20   604

>> size(sensor_data(2).p)

ans =

    10    10   604
```
